# Lower Trait Stability, Stronger Normative Beliefs, Habitual Phone Use, and Unimpeded Phone Access Predict Distracted College Student Messaging in Social, Academic, and Driving Contexts

**DOI:** 10.3389/fpsyg.2018.02633

**Published:** 2018-12-20

**Authors:** Julia L. Briskin, Tim Bogg, Jesse Haddad

**Affiliations:** Department of Psychology, Wayne State University, Detroit, MI, United States

**Keywords:** personality, messaging behavior, distraction and inattention, texting, phone use, structural equation modeling

## Abstract

The goal of the present study was to test two models of phone messaging behaviors among college students—a sociocognitive connection model and a cybernetic personality system model—across three contexts, where messaging behaviors represented disengagement from the primary context: a meal time with friends, attending class, and driving. Using a sample of university students (*N* = 634), path analyses with boot-strapping procedures were used to model direct and indirect effects of behavioral, social cognition, and personality trait predictors of primary context disengagement via message checking, message reading, and message sending behaviors. Internal and comparative model fit information showed the cybernetic personality system model represented the data well across all three contexts. Across the contexts, phone related habits and normative beliefs about phone usage mediated relations between personality traits and messaging behaviors. In addition, stronger normative beliefs for messaging behaviors and stronger phone related habits predicted unimpeded physical phone access across the contexts. Across contexts, more frequent messaging behaviors were most strongly predicted by the variance shared by low trait self-discipline, high trait anxiety, and high trait altruism via phone-related habits. The results are discussed in terms of the predictive utility of testing process models of messaging behaviors across varying contexts, as well as possible forms of intervention for reducing primary context disengagement via messaging behaviors.

## Introduction

Recent polls show 98% of young adults aged 18–29 own a mobile phone and 97% use mobile phones for text messaging (Duggan, 2013). While there are obvious benefits of mobile, handheld communication, mobile phone messaging behaviors may result in distracted or unsafe experiences across consequential contexts for young adults, including social interactions, educational settings, and operating automobiles (McEvoy et al., 2005; Drews et al., 2009; Wei et al., 2012; Przybylski and Weinstein, 2013; David et al., 2014; Misra et al., 2014). When phone messaging behaviors distract from engagement in these performance and social domains, possible consequences may include relationship dissatisfaction from friends, lower grades, and/or fatal automobile collisions. Given the possible negative sequelae of phone messaging behaviors that divert attention from a primary context or task, behavioral, and psychological researchers have begun developing and testing models of these potentially consequential and harmful behaviors.

The goal of the present study was to test and compare two process-oriented models of distracted mobile phone message checking, message reading, and message sending (collectively referred to as “messaging behaviors”) across social (eating with others), academic (being in class), and driving contexts using insights from models of (A) sociocognitive connection (Bayer et al., 2016), and (B) cybernetic (i.e., self-regulatory) personality systems (DeYoung, 2015). In the following sections, we review research on phone messaging behaviors and describe the components and organization of the sociocognitive connection and the cybernetic personality system models of primary context and task disengagement via messaging behaviors.

## Contexts of Messaging and Forms of Messaging Behaviors

While a substantial amount of research has investigated phone messaging behaviors, much of this research has focused on isolated contexts—messaging behaviors during social interactions (Przybylski and Weinstein, 2013; McDaniel and Coyne, 2016), messaging behaviors in classrooms (Wei et al., 2012; Lepp et al., 2015), or messaging behaviors while driving (Drews et al., 2009; Bayer and Campbell, 2012), but the degree to which the expression of these messaging behaviors may systematically vary *across* contexts has received considerably less attention. Additionally, research on messaging behaviors has typically focused on a single form of behavior, such as *sending* text messages, *reading* text messages, or *checking* text messages, rather than modeling messaging behaviors as indicators of an underlying behavioral expression.

The present study takes a systematic and integrative approach to contextual phone messaging behaviors, with the goal of explaining messaging behaviors across the contexts of eating with others, being in class, and driving. In order to advance an understanding of phone messaging behaviors across multiple contexts, two candidate models of messaging behaviors are reviewed in the following sections: a sociocognitive model of distracted messaging behaviors, and a cybernetic personality system model of distracted messaging behaviors.

### A Sociocognitive Connection Model of Distracted Messaging Behaviors

**Figure 1:**
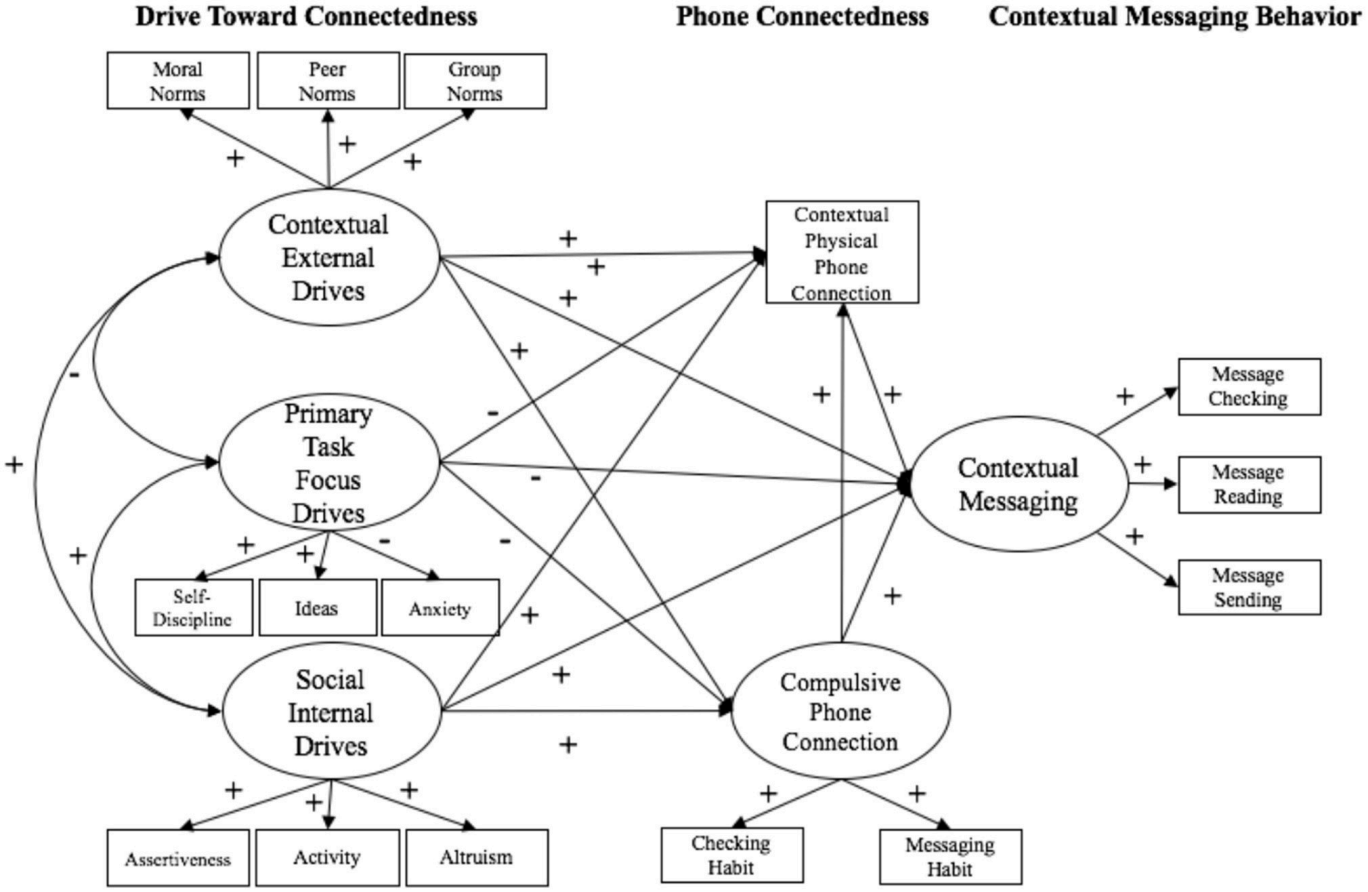
The sociocognitive connection model.

The sociocognitive perspective that can be applied to messaging behaviors is derived from three theoretical frameworks: (1) the social psychological theory describing a fundamental need to pursue and maintain meaningful interpersonal relationships (Baumeister and Leary, 1995); (2) The Theory of Planned Behavior, which proposes that perceived norms and attitudes play a role in predicting behavioral enaction (Ajzen, 1991); and (3) the social premise that, within wireless societies, connecting with others via mobile technology is expected, desired, and considered problematic if not achieved (Hall and Baym, 2012; Cheever et al., 2014; Bayer et al., 2016). In the sociocognitive connection model, a primary assumption is that phone users have a goal to connect with others using their mobile phones, and the extent to which this connection goal overtakes context-specific primary goals (i.e., eating a meal with a friend, paying attention in class, or driving) depends on connection norms, internal cues, and contextual cues (Bayer et al., 2016). In the adapted sociocognitive connection model proposed here, the extent to which a connection goal overtakes context-specific primary goals depends on external drives toward connectedness (i.e., connection norms), social internal drives and cues toward connectedness (i.e., dispositional tendencies that influence social connection), and the drive and ability to focus attention on primary goals (see Figure 1). These cues and drives predict mobile phone connectedness (i.e., physical phone location, compulsive phone connection), which, in turn, predicts messaging behaviors (i.e., message checking, message reading, and message sending).

#### Drive Toward Connectedness

As is depicted in Figure 1, the drive toward connectedness is determined by three sets of components: (1) external drives, including the perceived societal expectations to remain connected (group norms; Ajzen, 1991), peer group expectations to stay connected (peer norms), and the moral norms of connection behaviors (moral norms), (2) social internal drives, including the general tendency to place oneself in a social context (i.e., extraversion, specifically, the facets of activity, and assertiveness), and the general tendency to be amiable (i.e., agreeableness; specifically, the facet of altruism), and (3) the drive/ability to focus on a primary task or context. The drive to focus on a primary task or context is comprised of one's general tendency to be calm vs. anxious (i.e., neuroticism, and specifically, the facet of anxiety), the general tendency to be open to new experiences (i.e., openness; specifically, the facet of ideas), and the tendency to be responsible and orderly (i.e., conscientiousness; specifically, the facet of self-discipline). In line with Soto and John's (2009); Soto and John (2017) argument that trait facets (vs. the broader Big Five traits) can help increase utility and specificity for explaining behavior, trait facets were selected for inclusion in the proposed models. In the context of constructing explanatory models, the benefit of increased specificity of the trait facet scores affords the potential advantage of outweighing the “bandwidth” benefit that is traditionally associated with the Big Five personality traits (Soto and John, 2017).

External drives are proposed to encompass the societal (e.g., external) forces and accompanying social expectations that shape messaging beliefs and facilitate messaging habit formation, representing how social structures (e.g., norms) activate connection goals (Bayer et al., 2016). Social internal drives are proposed to encompass personality traits that reflect both the intrinsic desire to connect with others (i.e., extraversion; specifically, activity and assertiveness) and the propensity to cooperate with others when connecting (agreeableness; specifically, altruism). Previous research has linked extraversion with the “propensity to connect” with others (Totterdell et al., 2008; Bogg, 2017), and other research has shown that social approach and avoidance motivations are reflected by agreeableness, which ultimately serves the need to belong (Nikitin and Freund, 2008, 2014). The primary task focus drive is posited to reflect the dispositional tendencies that facilitate engagement with and attention to a primary goal. Research has shown that performance (i.e., career success; Judge et al., 1999) can be bolstered by lower neuroticism (specifically, low negative emotionality, reflected by low anxiety), greater openness (specifically intelligence, reflected by ideas), and greater conscientiousness (specifically, achievement orientation, reflected by self-discipline); thus, the primary task focus drive represents the combination of dispositional tendencies that afford persistent engagement with a primary task. Social internal and external drives toward connectedness are expected to be positively related to phone connectedness, while greater primary task focus drive is expected to be negatively related to phone connectedness.

#### Physical and Compulsive Phone Connectedness

Phone connectedness is comprised of (1) physical connectedness (i.e., location of one's mobile phone in proximity to the physical self in each context) and (2) compulsive connectedness (i.e., the habit strength related to messaging behaviors in any context). Recently, researchers have studied messaging behavior as a habit, theorizing that behavioral automaticity (habit strength) may be an independent predictor of the behavior (LaRose, 2010; Bayer and Campbell, 2012; Oulasvirta, 2012). Messaging might become habitual based on conditioned associations between drives toward connectedness and physical/compulsive phone connectedness. The related components of physical and compulsive connectedness are derived from Bayer and colleagues' model of connectedness, which suggests that physical phone messaging cues (audible sounds, visual signaling) and compulsive phone messaging cues (e.g., the time elapsed since the most recent connection with a social network and habit strength) ultimately catalyze phone messaging behaviors (Bayer et al., 2016).

### A Cybernetic Personality System Model of Distracted Messaging Behaviors

A second approach to modeling messaging behaviors is through a cybernetic framework of personality traits, in which behavior reflects a hierarchical set of control processes that serve values and goals (Powers, 1973). Models of interdependence among major personality traits have received increasing theoretical and empirical attention in recent years (Hirsh et al., 2009; Van Egeren, 2009; Bogg and Vo, 2014; DeYoung, 2015; Bogg, 2017). These models are derived from cybernetic feedback control theory, which describes how machines respond to various informational inputs to meet regulatory goals (Wiener, 1948; e.g., cruise control modulating speed in response to varying grades of road steepness). For personality adaptation, such models posit functions for major traits to enable adaptive actions that potentiate self-regulatory goals. For example, if a group norm for messaging availability is integrated into the self-regulatory processes of the personality system, then responses (e.g., increasing audible and visible phone alert settings vs. keeping a phone in an unobtrusive location) could be expected to vary in their presence and intensity based on individual differences in the dispositional drives for approach and engagement (i.e., extraversion, or being outgoing vs. inhibited).

Based on factor-analytic findings for the Big Five and recent theorizing, two meta-traits—stability and plasticity—are thought to represent superordinate mechanisms that monitor, control, and adapt system functioning to enable the actuation of regulatory goals (Digman, 1997; Hirsh et al., 2009; DeYoung, 2010). Stability represents components of neuroticism, conscientiousness, and agreeableness, providing means by which goal control, monitoring, and error detection related to system inputs (dynamic internal and external factors and circumstances) can be implemented. Plasticity represents components of extraversion and openness, providing means by which responses to system inputs can be implemented. These two meta-traits should differentially influence the perception of the environment (the input of the cybernetic loop), the self-regulatory goals (the reference value within the cybernetic loop), and the behavior that one engages to address a perceived discrepancy (the output of the cybernetic loop).

### Plasticity and Stability, Characteristic Adaptations, and On-Going Primary Context Engagement

**Figure 2:**
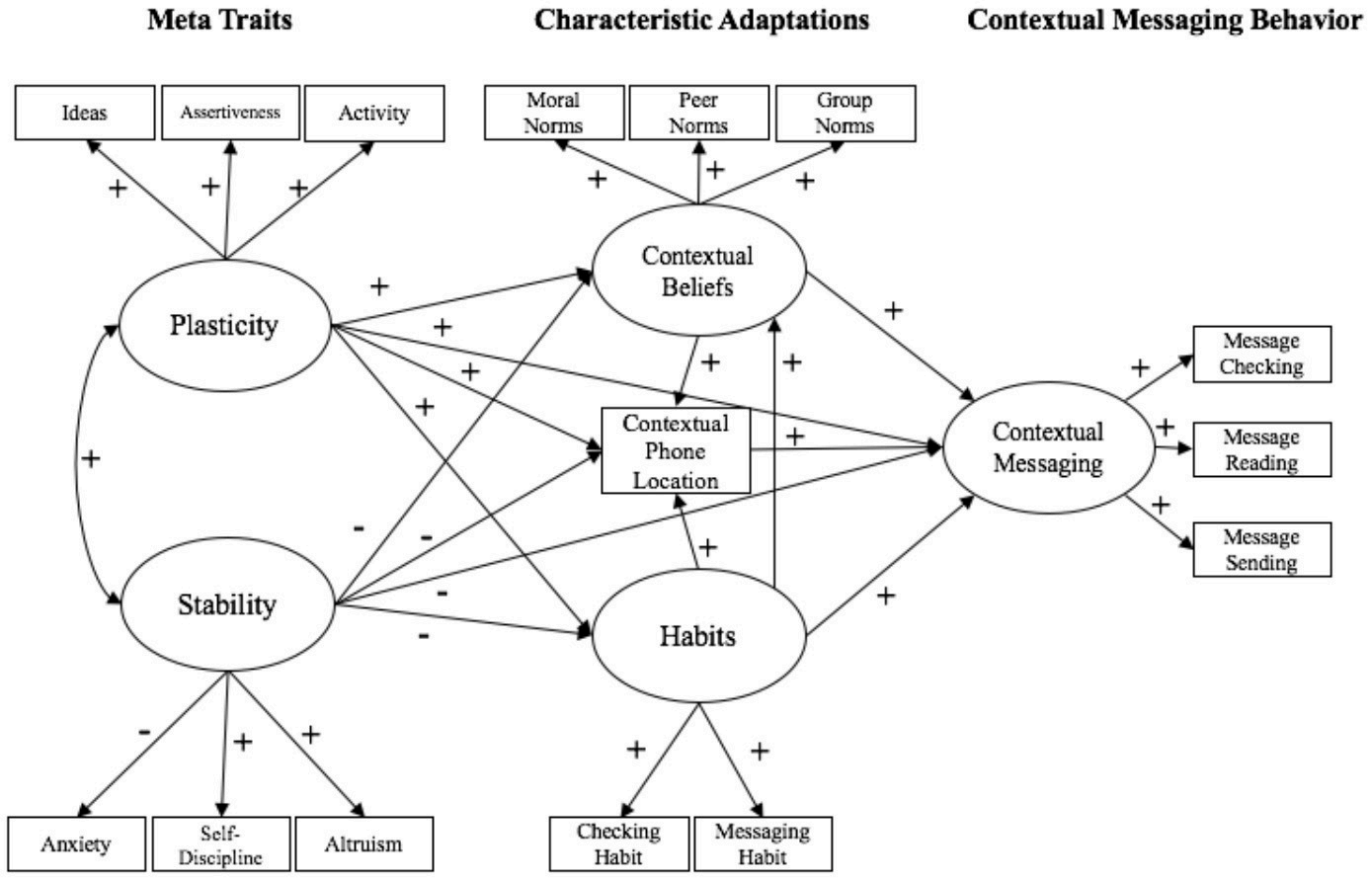
The cybernetic self-regulatory model.

As depicted in Figure 2, plasticity and stability are comprised of narrower self-regulatory personality trait drives. Specifically, greater plasticity reflects stronger approach and engagement drives (i.e., extraversion, specifically the facets of activity, and assertiveness), as well as sensory and information experiential drives (i.e., openness, specifically the facet of ideas). Greater stability reflects stronger drives to detect (i.e., neuroticism, specifically anxiety) and avoid (i.e., conscientiousness, specifically self-discipline) goal-related errors, as well as the coordination of goals with others (i.e., agreeableness, specifically altruism). Specific facets were selected for the proposed model to increase its explanatory power (Soto and John, 2017). While the traditional cybernetic rendering of personality includes the Big Five traits, specifying selected facets (which are conceptually distinct from their broader Big Five derivatives), is useful in maximizing the proposed model's explanatory power. Specifically, activity and assertiveness were both selected from extraversion, as both facets tap into approach (activity) and engagement (assertiveness) drives. The ideas facet was selected from openness over aesthetics, in line with the idea that experiential drives are more closely associated with thoughts rather than visual preferences (aesthetics). Anxiety was selected from neuroticism (rather than the depression facet) in line with the idea that goal-related errors are likely detected via hypervigilance, which is more closely associated with anxiety than depression. Self-discipline was selected over orderliness from conscientiousness, in line with the idea that self-discipline is more relevant to avoiding errors for goal selection (relative to orderliness). Finally, altruism was chosen over compliance from agreeableness, in line with the idea that caring about others (altruism) is more relevant to goal coordination than compliance. In a cybernetic rendering of major personality traits vis-à-vis plasticity and stability meta-traits, characteristic adaptations—semi-stable goals, interpretations, and strategies—are expected to be produced as a function of the combined regulatory influences of plasticity and stability in the context of environmental and situational affordances (see also McAdams and Pals, 2006; McCrae and Costa, 2008; DeYoung, 2015).

Greater tendencies for plasticity in engagement of primary contexts, as informed by greater approach and experiential drives, should be directly associated with more frequent expressions of specific forms of primary context disengagement (i.e., messaging behaviors). Moreover, greater plasticity should be indirectly associated with more frequent messaging behaviors via stronger related characteristic adaptations for messaging behaviors (i.e., greater endorsement of contextual beliefs, stronger behavioral habits, and unimpeded phone access). Greater tendencies for stability of engagement in primary contexts, characterized by goal-related error detection, avoidance, and coordinated control drives, should be directly associated with less frequent expressions of specific forms of primary context disengagement (i.e., messaging behaviors). Moreover, greater stability should be indirectly associated with less frequent messaging behaviors via weaker related characteristic adaptations for messaging behaviors (i.e., lesser endorsement of behavioral beliefs, weaker behavioral habits, and more impeded phone access).

## The Present Study

In the present study, a sociocognitive connection model and a cybernetic personality system model were used to examine distracted mobile phone messaging behaviors across three contexts (eating with others, being in class, and driving).

### Sociocognitive Connection Model Hypotheses

As depicted in Figure 1, across contexts, it was expected that individuals with greater internal and external drives to connect would have their phones more physically accessible (closer to one's person) and would have a stronger compulsion for use (indexed by greater habit strength). For individuals with greater primary task focus, it was expected that their phones would be less accessible, they would have a weaker compulsion for use, and they would engage in messaging behaviors less frequently. Moreover, it was expected that physical and compulsive phone connectedness would mediate, in part, associations between internal/external drives toward connectedness, internal drives and abilities for primary context focus, and messaging behaviors across contexts. From a sociocognitive connection perspective, the drive most relevant for a particular context should be more likely to be activated in that context, and should exert a stronger effect on messaging behaviors than drives less relevant to that context.

During a social interaction (i.e., eating a meal with others), when other people serve as goal activation cues, it was expected that external drives (beliefs) toward connectedness would be more salient and more strongly predictive of messaging behaviors than internal social drives or primary task focus drives, and would directly and indirectly (via both physical and compulsive phone connectedness) positively predict messaging behaviors. Social internal drives to connect were expected to show a similar but weaker pattern of effects, as one's social goals are likely to be at least partially fulfilled in the context of eating with others, thereby reducing the necessity of using one's phone to fulfill social goals. Moreover, when external drives (beliefs about normative phone use) are salient, it was expected that conforming to social norms and adhering to peer expectations would seem more important (i.e., attending to the meal-time partner), and internal primary task focus drives would be activated for regulating (and preventing) distracted messaging behaviors. Thus, it was predicted that primary task focus drive would negatively predict messaging behaviors in the context of eating with others, and would indirectly predict messaging behaviors via a reduction of both physical and compulsive phone connectedness.

In a classroom context, it was expected that primary task focus drives would exert the strongest direct effect, negatively predicting messaging behaviors, as well as indirectly predicting reduced messaging behaviors via lesser phone connectedness. It was also expected that greater primary task focus would lead to decreased compulsive phone connection, as well as less physical phone connection (i.e., more distal phone location), both of which were posited to positively predict messaging behaviors. Moreover, it also was expected that internal and external drives would both directly and indirectly positively predict messaging behaviors in class via phone connectedness (phone location and compulsive phone readiness), but would exert weaker effects than primary task focus drives. Both internal and external drives to connect were expected to be equally predictive in a classroom setting; norms may be activated as others use their mobile phones in class, and internal social drives, such as extraversion, may also be activated as goals to pursue sociality remain unfulfilled in a classroom lecture setting.

In the context of driving, internal drives and primary task focus drives should exert particularly strong direct effects on messaging behaviors, while external drives should exert relatively little direct influence on messaging behaviors. The premise for this hypothesis is that driving is largely a solitary activity, and external drives would be less salient in the absence of other people (who serve as cues for activating external drives). However, in the absence of others, internal cues (such as the desire to interact with others, i.e., extraversion) and tendencies (i.e., one's tendency to be self-disciplined) may be more motivationally salient and, thus, will exert stronger effects on messaging behaviors than external drives. It was expected that internal social drives would positively predict messaging behaviors and would positively predict phone connectedness (both compulsive phone connectedness and physical phone connectedness), which would, in turn, positively predict messaging behaviors. It was also expected that primary task focus drives would directly negatively predict messaging behaviors in a driving context, and primary task focus drives would indirectly negatively predict messaging behaviors via reduced physical and compulsive phone connectedness while driving.

### Cybernetic Personality System Model Hypotheses

As depicted in Figure 2, it was expected that individuals with greater plasticity would report greater primary context disengagement via messaging behaviors. Conversely, it was expected that individuals with greater stability would report less primary context disengagement via messaging behaviors. It also was expected that individuals with greater plasticity would have more positive normative beliefs toward messaging behaviors within each context, stronger phone use habits, and would have their phones more readily available to them. By contrast, it was expected that individuals with greater stability would have more negative beliefs toward messaging behaviors within each context, have weaker phone messaging habits, and would have their phones less readily available to them. Moreover, it was expected that characteristic adaptations (i.e., normative beliefs, phone accessibility, and messaging habits) would, in part, maintain the associations between plasticity and stability and messaging behaviors across contexts.

Given the nature of the drives for plasticity and stability, stronger effects were expected for drives and characteristic adaptations that could not easily be satisfied in a given context. Specifically, greater plasticity and associated characteristic adaptations were expected to show stronger effects in the classroom and driving settings, where approach, and to a lesser extent, experiential drives are constrained and phone messaging provides a means (when further catalyzed by beliefs, habits, and readiness) to satisfy the drives for engagement and stimulation. By comparison, plasticity and related characteristic adaptation effects were expected to be present, but weaker, in the context of eating with others, where approach and engagement drives, at least in principle, can be readily satisfied. Greater stability and associated characteristic adaptations were expected to show stronger effects in the classroom and driving settings, where goal-related error detection, avoidance, and control drives should be particularly sensitive to deviations from primary task engagement, which requires persistent in-the-moment attentiveness. By comparison, stability and related characteristic adaptation effects were expected to be present, but weaker, in the context of eating with others, where again, at least in principle, task engagement does not require the consistent application of attention.

## Methods

### Participants

Participants were undergraduate students from Wayne State University. There were two phases of data collection: one phase of data collection took place in the lab (*N* = 295), and the second phase of data collection took place online (*N* = 522). The protocol and materials were approved with exempt status by Wayne State University's Behavioral Institutional Review Board; to minimize the risk of breaching confidentiality, written informed consent was not obtained, but participants verbally (or electronically) indicated their willingness to participate in the study after reading a study information sheet. Students received research credits for psychology courses via a psychology department research participation recruitment system. Eight online participants took the survey twice and had different responses in each survey, thus data from those 16 surveys was removed. Thirty-one additional participants were excluded for failing online attention checks. Sixty-nine participants were excluded for spending <10 min on the online survey (which was the minimum amount of time that the online survey reasonably took to complete). To allow for bootstrapping procedures, missing data were removed, rather than imputed (54 participants; 27 from in lab data and 27 from online data).

There were 4 univariate outliers, but differences in scores between outliers and non-outliers were extremely modest, so they were included in analyses. Thirteen multivariate outliers were removed to satisfy the assumption of multivariate normality for structural equation modeling. We assumed extreme combinations of scores reflected individuals from a population that we did not intend to sample. An examination of univariate outliers revealed some individuals were considerably older than the typical college student (i.e., over the age of 40), and age has been considered a relevant factor for explaining one's willingness to use technology (i.e., one's perceptions of technology; Vaportzis et al., 2017). Older adults tend to perceive more barriers to technology compared to younger individuals (i.e., lack of instructional support; Vaportzis et al., 2017), and we wanted to avoid confounding our analysis by including individuals who did not belong to the target sample. Thus, in line with Kline's 2011 recommendations, we removed the 13 individuals who were assumed to be outside of the traditional scope of a college sample. The final sample consisted of 634 participants (*M*_*age*_ = 21.19 years, *SD*_*age*_ = 4.77 years). The sample contained more female than male participants (31.9% males), and was ethnically diverse (41.1% Caucasian/European American, 16.1% Asian American, 17.5% African American/Black, 9.5% Arabic/Middle Eastern, 4.7% Hispanic/Chicano/Mexican American, 0.3% Native American, and 10.7% other or multiple ethnicities). Participants were primarily single (61.6% single, 33.7% in a committed relationship, 3.7% married, 0.2% separated, and 1.0% divorced), and most participants did not have children (94.5%). More than half of the student participants were currently employed (61.5% currently working). In order to assess differences on key variables of interest between the in-lab participants and the online participants, mean difference tests were conducted. Results showed that in-lab participants reported phone behavior as significantly more normative than online participants (for peer and moral norms in the context of driving, and moral norms for the context of eating). In addition, in-lab participants reported being significantly farther from their phones during class, and reported significantly weaker phone checking and messaging habits compared to the online participants. No other mean difference tests were significant at *p* < 0.001. Partial eta squared ranged from 0.03 to 0.07 for significant differences, suggesting that these statistically significant differences were not practically meaningful.

### Procedure

Participants were first instructed to read a study information sheet after which a research assistant briefly explained the study and the format of the assessment session (for in-lab participants). In-lab participants were directed to a private assessment room and were provided with instructions for the completion of the questionnaires (described below), and online participants completed the questionnaires via a secure Qualtrics survey link. During the instructions, participants were shown different response scales for the questionnaires and reminded that all answers were to be based on actual experience rather than what might be desirable. Participants were then instructed to complete the questionnaires and were encouraged to ask questions for clarification; for online participants, a text box was provided at the end of the survey to document any questions/comments that participants had. Research staff returned to the in-lab assessment rooms in 15 min intervals to ascertain progress and answer questions. The questionnaires required 45–60 min to complete in the lab, and 10–60 min online (the mean online completion time was 24 min).

## Assessment Materials

### Contextual Phone Behaviors

The dependent variables of interest were frequency of message checking, message reading, and message sending behaviors across the contexts of eating with others, being in class, and driving. The questionnaires assessed the frequency of these three messaging behaviors across the three contexts, totaling nine questions. This questionnaire was an expanded version of the texting while driving questionnaire used by Bayer and Campbell (2012). Mobile phone messaging behavior was defined as “text messaging, instant messaging (e.g., Facebook mobile messenger), Snapchatting, and other forms of mobile social media messaging,” in order to take into account other common forms of messaging. Phone message checking was defined as “briefly activating and viewing the home screen to check for new messages or other notifications without being prompted by vibration or sound.” The contexts “eating with others” and “in class” were chosen (as opposed to “spending time with others” or “doing homework/studying”) because they were more specific and time-restrained, thus making it more likely for participants to accurately recall behaviors. All questions used a 5-point response scale (1 = “Almost Never” to 5 = “Almost Always”).

### Context Specific Phone Accessibility

Phone accessibility was determined by the reported typical physical location of one's mobile phone in each context (i.e., in sight with the screen facing up = 1, in sight with the screen facing down = 2, in one's pocket = 3, in one's purse/bag = 4, or elsewhere/inaccessible = 5), using a five-point scale, which was reverse-coded before analyses were conducted, so that larger values indicated greater phone accessibility. Phone accessibility functioned as a component of phone connectedness in the sociocognitive connection model, and was a component of characteristic adaptations for the cybernetic personality system model.

### Frequency-Independent Self-Report Habit Index

A 12-item habit scale was used to assess habit strength of phone messaging behavior (α = 0.93) and phone checking behavior (α = 0.93), independent of behavioral frequency (Orbell and Verplanken, 2010; Bayer and Campbell, 2012). This scale is used to assess the habitual and automatic qualities of phone messaging and phone checking behaviors (cf. Bayer and Campbell, 2012), which have been found to be often habituated (Oulasvirta, 2012). Items included “phone messaging/checking is something I do automatically” and “phone messaging/checking is something that would require effort not to do.” This measure used a 7-point Likert scale (1 = “Disagree Strongly” to 7 = “Agree Strongly”). This measure was a component of compulsive phone connectedness in the sociocognitive model and was a component of habits in the cybernetic personality system model.

### Theory of Planned Behavior (TPB) Measures

Three separate TPB questionnaires were used for each of the three contexts, consisting of the same sets of questions adapted from Bayer and Campbell (2012). All items used a 7-point Likert scale (1 = “Disagree Strongly” to 7 = “Agree Strongly”). Perceived peer norms for mobile phone messaging behaviors were assessed using two items (i.e., “My friends would think it is inappropriate to read or send messages,” reverse scored, and “My friends would approve of me reading or sending messages”) across three separate contexts: while eating with others (peer norms: α = 0.63), while in class (peer norms: α = 0.54), and while driving (peer norms: α = 0.51).

Group norms were assessed using three items (i.e., “People who I look up to would approve of me reading or sending messages,” “People who are important to me would think it is okay to read or send messages,” and “People who I respect would think it is appropriate to read or send messages”) across the same three contexts: while eating with others (group norms: α = 0.87), while in class (group norms: α = 0.93), and while driving (group norms: α = 0.88).

The three reverse-scored items for moral norms included “I would feel guilty if I read or sent messages,” “I personally think that it is wrong to read or send messages,” and “It goes against my principles to read or send messages” across the same three contexts: while eating with others (moral norms: α = 0.88), while in class (moral norms: α = 0.90), and while driving (moral norms: α = 0.85). All items were scored so that higher scores reflected more positive views of mobile phone messaging behaviors across three contexts. Peer, group, and moral norms were components of the external drives to connect in the sociocognitive connection model, and part of contextual beliefs in the cybernetic personality system model.

### Personality Trait Scales

The Big Five Inventory was used to measure both the Big Five personality traits and their facets: extraversion (α = 0.84) with assertiveness (α = 0.75) and activity (α = 0.72) as facets, agreeableness (α = 0.76) with altruism (α = 0.57) and compliance (α = 0.53) as facets, conscientiousness (α = 0.73) with orderliness (α = 0.50) and self-discipline (α = 0.55) as facets, neuroticism (α = 0.83) with anxiety (α = 0.75), and depression (α = 0.52) as facets, and openness (α = 0.73) with aesthetics (α = 0.56) and ideas (α = 0.55) as facets (BFI: John et al., 1991; 2008). Selected facet scales, rather than the global trait scales, were used for the primary analyses to improve the conceptual specificity of the analyses for messaging behaviors using the two process models described above (cf. Paunonen and Ashton, 2001). Specifically, the self-discipline facet of conscientiousness (e.g., “I am someone who perseveres until the task is finished”), the ideas facet of openness (e.g., “I am someone who is curious about many different things”), and the anxiety facet of neuroticism (e.g., “I am someone who worries a lot”) were components of primary task focus drives in the sociocognitive model, while the activity and assertiveness facets of extraversion (e.g., “I am someone who is full of energy” for activity and “has an assertive personality” for assertiveness), and the altruism facet of agreeableness (e.g., “I am someone who is helpful and unselfish with others”) were components of social internal drives. For the cybernetic model, the personality facets of extraversion (activity and assertiveness), and openness (ideas), were components of plasticity, while neuroticism (anxiety), conscientiousness (self-discipline), and agreeableness (altruism) were components of stability. Facets with greater theoretical relevance and reliabilities were selected for inclusion in the models.

## Analyses

Correlational analyses were used to ascertain the size and directionality of bivariate effects. Path models for the sociocognitive connection perspective and the cybernetic personality system perspective were then constructed and analyzed using Amos (v. 23.0)—precisely as depicted in Figures 1 and 2, respectively. Three sets of the sociocognitive connection model and three sets of the cybernetic personality system model were analyzed to predict messaging behaviors in the contexts of eating with others, being in class, and driving. Models were tested using the personality trait facets described above^1^. However, the initial measurement models showed that the “ideas” facet did not adequately reflect the hypothesized latent constructs of primary task focus drive from the sociocognitive connection perspective or plasticity from the cybernetic personality system perspective. As a result, the ideas facet was removed from all subsequent analyses.

For the sociocognitive connection model, latent variables were created for each component of the model (see Figure 1). Peer, group, and moral norms (using items from the Theory of Planned Behavior measures), served as indicators for the latent construct of external drives toward connectedness, reflecting societal expectations for connection. The facets of assertiveness, activity, and altruism served as indicators for the latent construct of social internal drives toward connectedness, reflecting individual dispositional tendencies to connect and cooperate with others. The self-discipline and anxiety facets of the BFI served as indicators for primary task focus drives, reflecting the combination of dispositional tendencies that facilitate attention to a primary task. The phone accessibility item (indexed by phone location) was included as a manifest variable that represented context-specific physical phone connectedness. The two items corresponding to phone checking and phone messaging habits served as indicators for trans-context compulsive phone connectedness. A latent construct of context-specific messaging behaviors was created from the contextual phone behavior scale, indicated by message checking, message reading, and message sending behaviors.

For the cybernetic personality system model, latent constructs were again created for each component in the model (see Figure 2). The assertiveness and activity facets of the BFI served as indicators for plasticity, and the anxiety, self-discipline, and altruism facets of the BFI were used as indicators for stability. These selected facets reflect the traits that comprise plasticity and stability, according to cybernetic theory (DeYoung, 2010). Theory of Planned Behavior scale items for peer, group, and moral norms served as indicators for contextual beliefs. The phone accessibility item was a manifest variable representing context-specific behavioral readiness. The two items corresponding to phone checking and phone messaging habits were used as indicators of phone habits. As in the sociocognitive model, a latent construct of context-specific messaging behaviors was created from the contextual phone behavior scale, indicated by message checking, message reading, and message sending behaviors.

The root mean square error of approximation (RMSEA) and the comparative fit index (CFI) were used to assess internal model fit. RMSEA is used to index the discrepancy between the observed covariance matrix (in relation to its degrees of freedom) and the hypothesized covariance matrix (Cangur and Ercan, 2015); values of 0.01, 0.05, and 0.08 represent excellent, good, and mediocre fits, respectively (MacCallum et al., 1996). The CFI is a “goodness of fit” statistic, ranging from 0 to 1, indicating the extent to which the model of interest is better at reproducing the observed data relative to the independence model (a model which assumes that variables are unrelated). CFI scores over 0.93 are considered acceptable (Byrne, 1994). RMSEA and CFI are less sensitive to sample size than other fit indices (Fan et al., 1999).

Model comparisons were made using the Akaike information criterion (AIC) and the Browne-Cudeck criterion (BCC). These are “information theory goodness of fit measures” that indicate which model (or models) best reproduce the observed variance/covariance matrices using the fewest possible parameters. Smaller AIC and BCC values correspond to comparatively better fitting models (Akaike, 1987); the BCC penalizes model complexity to a greater degree than the AIC, thus, both are reported.

## Results

### Correlational Analyses

Tables 1–3 display descriptive statistics and correlations for the variables under study, across the contexts of eating with others, being in class, and driving. Of particular interest, phone checking and messaging habits, as well as peer, group, and moral norms were significantly positively correlated with message checking, reading, and sending behaviors across contexts. Self-discipline and ideas (facets of conscientiousness and openness, respectively), were significantly negatively correlated with message checking, reading, and sending behaviors only in the contexts of eating with others and being in class; assertiveness was significantly positively correlated with message checking, reading, and sending behaviors only in the context of driving.

**Table 1:** Descriptive statistics and bivariate correlations for the context of eating with others.

	***M***	***(SD)***	**1**	**2**	**3**	**4**	**5**	**6**	**7**	**8**	**9**	**10**	**11**	**12**	**13**	**14**	**15**
1. Assertiveness	14.47	4.38															
2. Activity	7.50	1.89	0.521^**^	.													
3. Altruism	16.49	2.60	0.058	0.247^**^	.												
4. Self-Discipline	17.99	3.14	0.092^*^	0.213^**^	0.268^**^	.											
5. Anxiety	12.42	3.67	−0.179^**^	−0.266^**^	−0.113^**^	−0.212^**^	.										
6. Ideas	18.48	2.83	0.164^**^	0.305^**^	0.146^**^	0.115^**^	−0.133^**^	.									
7. Moral norms	10.91	4.54	−0.011	−0.027	−0.127^**^	−0.060	−0.090^*^	−0.111^**^	.								
8. Peer norms	9.10	2.79	−0.018	−0.002	−0.065	−0.024	−0.018	−0.009	0.410^**^								
9. Group norms	9.09	4.29	−0.047	0.011	−0.097^*^	0.019	−0.098^*^	−0.028	0.372^**^	0.347^**^							
10. Phone location	3.56	1.05	0.008	0.002	−0.082^*^	−0.045	0.079^*^	−0.102^*^	0.268^**^	0.185^**^	0.160^**^						
11. Checking habit	4.82	1.39	0.010	−0.023	0.011	−0.105^**^	0.216^**^	−0.114^**^	0.043	0.058	0.020	0.210^**^					
12. Messaging habit	4.19	1.46	0.121^**^	0.102^*^	0.001	−0.059	0.102^*^	−0.091^*^	0.010	0.052	0.075	0.207^**^	0.621^**^				
13. Message check	2.98	1.16	0.029	0.031	−0.075	−0.103^**^	0.080^*^	−0.121^**^	0.356^**^	0.231^**^	0.179^**^	0.402^**^	0.484^**^	0.398^**^			
14. Message reading	3.03	1.15	−0.023	−0.032	−0.084^*^	−0.103^**^	0.059	−0.153^**^	0.425^**^	0.263^**^	0.204^**^	0.372^**^	0.359^**^	0.325^**^	0.770^**^		
15. Message sending	2.80	1.18	0.008	0.016	−0.061	−0.115^**^	0.085^*^	−0.116^**^	0.395^**^	0.245^**^	0.231^**^	0.386^**^	0.376^**^	0.385^**^	0.769^**^	0.830^**^	.

** Indicates statistical significance at the 0.01 level (2-tailed) and

* *indicates statistical significance at the 0.05 level (2-tailed)*.

**Table 2:** Descriptive statistics and bivariate correlations for the context of being in class.

	***M***	***(SD)***	**1**	**2**	**3**	**4**	**5**	**6**	**7**	**8**	**9**	**10**	**11**	**12**	**13**	**14**	**15**
1. Assertiveness	14.47	4.38	.														
2. Activity	7.50	1.89	0.521^**^	.													
3. Altruism	16.49	2.60	0.058	0.247^**^	.												
4. Self-discipline	17.99	3.14	0.092^*^	0.213^**^	0.268^**^	.											
5. Anxiety	12.42	3.67	−0.179^**^	−0.266^**^	−0.113^**^	−0.212^**^	.										
6. Ideas	18.48	2.83	0.164^**^	0.305^**^	0.146^**^	0.115^**^	−0.133^**^	.									
7. Moral norms	10.91	4.54	0.053	0.005	0.010	−0.072	−0.029	−0.071									
8. Peer norms	9.10	2.79	0.056	−0.033	0.038	−0.049	0.011	0.022	0.474^**^								
9. Group norms	9.09	4.29	0.010	0.017	−0.009	−0.061	0.002	−0.015	0.287^**^	0.280^**^							
10. Phone location	3.56	1.05	0.075	0.054	0.006	−0.028	0.007	−0.051	0.289^**^	0.192^**^	0.148^**^						
11. Checking habit	4.82	1.39	0.010	−0.023	0.011	−0.105^**^	0.216^**^	−0.114^**^	0.126^**^	0.138^**^	0.032	0.171^**^					
12. Messaging habit	4.19	1.46	0.121^**^	0.102^*^	0.001	−0.059	0.102^*^	−0.091^*^	0.060	0.086^*^	0.078	0.228^**^	0.621^**^				
13. Message check	3.25	1.16	0.027	−0.001	−0.061	−0.157^**^	0.096^*^	−0.130^**^	0.352^**^	0.233^**^	0.157^**^	0.445^**^	0.426^**^	0.361^**^			
14. Message reading	3.33	1.16	0.065	0.051	−0.027	−0.150^**^	0.083^*^	−0.129^**^	0.356^**^	0.212^**^	0.143^**^	0.456^**^	0.399^**^	0.376^**^	0.831^**^		
15. Message sending	3.12	1.21	0.065	0.029	−0.001	−0.145^**^	0.105^**^	−0.124^**^	0.365^**^	0.257^**^	0.178^**^	0.458^**^	0.397^**^	0.384^**^	0.791^**^	0.858^**^	.

** Indicates statistical significance at the 0.01 level (2-tailed) and

* *indicates statistical significance at the 0.05 level (2-tailed)*.

**Table 3:** Descriptive statistics and bivariate correlations for the driving context.

	***M***	***(SD)***	**1**	**2**	**3**	**4**	**5**	**6**	**7**	**8**	**9**	**10**	**11**	**12**	**13**	**14**	**15**
1. Assertiveness	14.47	4.38	.														
2. Activity	7.50	1.89	0.521^**^	.													
3. Altruism	16.49	2.60	0.058	0.247^**^	.												
4. Self-Discipline	17.99	3.14	0.092^*^	0.213^**^	0.268^**^	.											
5. Anxiety	12.42	3.67	−0.179^**^	−0.266^**^	−0.113^**^	−0.212^**^	.										
6. Ideas	18.48	2.83	0.164^**^	0.305^**^	0.146^**^	0.115^**^	−0.133^**^	.									
7. Moral norms	10.91	4.54	0.127^**^	0.043	−0.105^**^	−0.097^*^	−0.141^**^	−0.041									
8. Peer norms	9.10	2.79	0.032	−0.003	−0.148^**^	−0.069	−0.093^*^	0.011	0.324^**^								
9. Group norms	9.09	4.29	0.009	0.006	−0.186^**^	−0.076	−0.097^*^	−0.012	0.300^**^	0.353^**^							
10. Phone location	3.56	1.05	0.135^**^	0.046	−0.029	−0.011	−0.009	0.055	0.243^**^	0.123^**^	0.052						
11. Checking habit	4.82	1.39	0.010	−0.023	0.011	−0.105^**^	0.216^**^	−0.114^**^	0.050	0.041	−0.023	0.165^**^					
12. Messaging habit	4.19	1.46	0.121^**^	0.102^*^	0.001	−0.059	0.102^*^	−0.091^*^	0.042	0.028	0.067	0.088^*^	0.621^**^				
13. Message check	2.18	1.13	0.146^**^	0.031	−0.035	−0.108^**^	0.025	0.029	0.394^**^	0.168^**^	0.233^**^	0.412^**^	0.322^**^	0.286^**^			
14. Message reading	2.29	1.21	0.136^**^	0.043	−0.011	−0.106^**^	0.020	0.005	0.410^**^	0.195^**^	0.176^**^	0.421^**^	0.294^**^	0.279^**^	0.786^**^		
15. Message sending	1.98	1.09	0.171^**^	0.084^*^	0.013	−0.091^*^	0.014	0.058	0.412^**^	0.181^**^	0.168^**^	0.421^**^	0.274^**^	0.299^**^	0.761^**^	0.830^**^	.

** Indicates statistical significance at the 0.01 level (2-tailed) and

* *indicates statistical significance at the 0.05 level (2-tailed)*.

### Comparison of the Sociocognitive Connection and the Cybernetic Personality System Models Across Contexts

Table 4 shows the fit statistics for the sociocognitive connection model and the cybernetic model for each context. The sociocognitive connection model had adequate fit for both the eating with others and driving contexts, and good fit for the context of being in class. CFI scores for the eating with others, in class, and driving contexts, respectively, indicated that ~95, 97, and 94% of the covariation in the data was reproduced by the sociocognitive connection model. RMSEA scores of 0.06, 0.05, and 0.06 for the eating with others, in class, and driving contexts, respectively, indicated that error of approximation was acceptable in relation to the model's degrees of freedom.

**Table 4:** Fit statistics for the sociocognitive connection model and the cybernetic personality system model.

**Retained model name/Context**	**Internal fit indices**					**Comparative fit Indices**	
	**X^**2**^**	***df***	***p***	**RMSEA**	**CFI**	**AIC**	**BCC**
Sociocognitive eating context	197.41	64	0.001	0.06	0.95	307.41	310.08
Cybernetic eating context	196.20	64	0.001	0.06	0.95	306.20	308.87
Sociocognitive class context	147.60	64	0.001	0.05	0.97	257.60	260.27
Cybernetic Class Context	140.39	64	0.001	0.04	0.98	250.39	253.06
Sociocognitive driving context	229.66	64	0.001	0.06	0.94	339.66	342.33
Cybernetic driving context	229.03	64	0.001	0.06	0.94	339.03	341.70
**ALTERNATIVE MODELS (FACET SCORES)**							
Sociocognitive eating context	229.29	76	0.001	0.06	0.95	347.29	350.35
Cybernetic eating context	219.29	76	0.001	0.06	0.95	337.72	340.78
Sociocognitive class context	180.27	76	0.001	0.05	0.97	298.28	301.34
Cybernetic class context	163.94	76	0.001	0.04	0.97	281.94	285.00
Sociocognitive driving context	257.20	76	0.001	0.06	0.94	365.20	378.26
Cybernetic Driving Context	255.95	76	0.001	0.06	0.94	373.95	377.01
**ALTERNATIVE MODELS (GLOBAL TRAITS)**							
Sociocognitive eating context	N/A	N/A	N/A	N/A	N/A	N/A	N/A
Cybernetic eating context	245.72	63	0.001	0.07	0.93	357.72	360.44
Sociocognitive class context	N/A	N/A	N/A	N/A	N/A	N/A	N/A
Cybernetic class context	N/A	N/A	N/A	N/A	N/A	N/A	N/A
Sociocognitive driving context	N/A	N/A	N/A	N/A	N/A	N/A	N/A
Cybernetic driving context	267.36	63	0.001	0.07	0.92	379.36	382.07

*p < 0.001. The letters “N/A” indicate instances in which models failed to converge*.

The cybernetic model also had adequate fit for both the eating with others and driving contexts, and good fit for the context of being in class. The CFI scores indicated that ~95, 98, and 94% of the covariation in the data was reproduced by the cybernetic model for the contexts of eating with others, being in class, and driving, respectively. RMSEA scores of 0.06, 0.04, and 0.06, for the contexts of eating with others, being in class, and driving, respectively, indicated that error of approximation was acceptable in relation to the model's degrees of freedom. To summarize, the internal model indices suggest each model showed adequate to good levels of discrepancy between the observed covariance matrices (in relation to its degrees of freedom) and the hypothesized covariance matrices for each context, and both models adequately reproduced the observed data relative to the independence model (see Table 4). However, the cybernetic model had slightly better internal fit for all three contexts.

As indicated by the AIC and BCC, the cybernetic model fit moderately better than the sociocognitive connection model for the context of being in class, however neither model was preferred for the contexts of eating with others or driving (see Table 4). An examination of the specific pathways in each model suggested that the cybernetic model represented the data more parsimoniously than the sociocognitive connection model. Specifically, the effects of Plasticity and Stability on messaging behavior via habits were strong and consistent across contexts, and in line with our theoretical analysis. By contrast, pathways in the sociocognitive connection model were more diffuse, requiring greater model complexity without providing additional explanatory power. To summarize, the comparative model fit indices suggest the cybernetic personality system model best reproduced the observed variance/covariance matrices using the fewest possible parameters for all three contexts. As a result, the cybernetic model was selected for interpretation across all three contexts.

**Figure 3:**
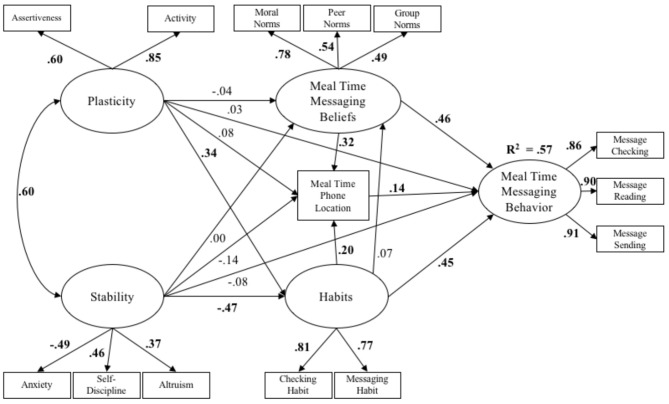
The cybernetic personality system model in the meal time context (*N* = 634). All standardized path coefficients that were significant at the 0.05 level are bolded.

Figure 3 displays the standardized path coefficients for the cybernetic model in the context of eating with others. Plasticity and stability were significantly positively correlated. Greater plasticity indirectly predicted increased messaging behaviors via increased phone-related habits. Stability did not directly predict messaging behaviors, however, stability indirectly predicted a reduction in messaging behaviors via decreased phone related habits. Normative beliefs about messaging behaviors, physical phone location, and phone-related habits directly predicted increased messaging behaviors, and both normative beliefs about messaging behaviors and phone-related habits indirectly positively predicted messaging behaviors via physical phone location. Total, direct, and indirect effects, as well as bias corrected bootstrapped standard errors and 95% confidence intervals are displayed in Table 5.

**Table 5:** Standardized total, direct, and indirect effects, standard errors, and 95% confidence intervals for contextual messaging behavior for the cybernetic personality system model.

**Context**	**Plasticity**	**Stability**	**Habits**	**Contextual normative beliefs**	**Contextual phone location**
**Eating (Cybernetic)**	**β (SE) (95 % CI)**	**β (SE) (95 % CI)**	**β (SE) (95 % CI)**	**β (SE) (95 % CI)**	**β (SE) (95 % CI)**
Total Effect	0.20^*^ (0.11)	−0.33^**^ (0.12)	0.51^**^ (0.06)	0.51^**^ (0.04)	0.14^**^ (0.04)
	(0.04, 0.49)	(−0.65, −0.14)	(0.40, 0.62)	(0.43, 0.59)	(0.07, 0.22)
Direct Effect	0.03 (0.08)	−0.08 (0.10)	0.50^**^ (0.05)	0.46^**^ (0.04)	0.14^**^ (0.04)
	(−0.11, 0.21)	(−0.29, 0.11)	(0.35, 0.54)	(0.37, 0.54)	(0.07, 0.22)
Indirect Effect	0.17^*^ (0.08)	−0.26^**^ (0.09)	0.07 (0.05)	0.05^**^ (0.01)	N/A
	(0.03, 0.36)	(−0.50, −0.11)	(−0.02, 0.16)	(0.02, 0.08)	N/A
**CLASS (CYBERNETIC)**					
Total Effect	0.29^**^ (0.15)	−0.41^**^ (0.16)	0.48^**^ (0.16)	0.39^**^ (0.05)	0.29^**^ (0.07)
	(0.12, 0.56)	(−0.71, −0.22)	(0.36, 0.59)	(0.30, 0.46)	(0.21, 0.36)
Direct Effect	0.12 (0.93)	−0.19^*^ (1.00)	0.36^**^ (0.28)	0.29^**^ (0.06)	0.29^**^ (0.07)
	(−0.02, 0.34)	(−0.42, −0.01)	(0.22, 0.45)	(0.20, 0.38)	(0.21, 0.36)
Indirect Effect	0.18^**^ (0.83)	−0.22^**^ (0.89)	0.12^**^ (0.13)	0.09^**^ (0.03)	N/A
	(0.06, 0.31)	(−0.39, −0.09)	(0.06, 0.18)	(0.06, 0.13)	N/A
**DRIVING (CYBERNETIC)**					
Total Effect (SE)	0.27^**^ (0.09)	−0.27^**^ (0.11)	0.37^**^ (0.06)	0.53^**^ (0.06)	0.28^**^ (0.04)
	(0.13, 0.52)	(−0.61, −0.10)	(0.25, 0.47)	(0.41, 0.63)	(0.20, 0.36)
Direct Effect (SE)	0.02 (0.09)	0.00 (0.13)	0.33^**^ (0.06)	0.46^**^ (0.06)	0.28^**^ (0.04)
	(−0.16, 0.20)	(−0.23, 0.28)	(0.21, 0.44)	(0.33, 0.56)	(0.20, 0.36)
Indirect Effect (SE)	0.24^**^ (0.09)	−0.28^*^ (0.13)	0.04 (0.05)	0.08^**^ (0.02)	N/A
	(0.08, 0.53)	(−0.57, −0.05)	(−0.07, 0.13)	(0.05, 0.12)	N/A

p < 0.05^*^, p < 0.01^**^.

**Figure 4:**
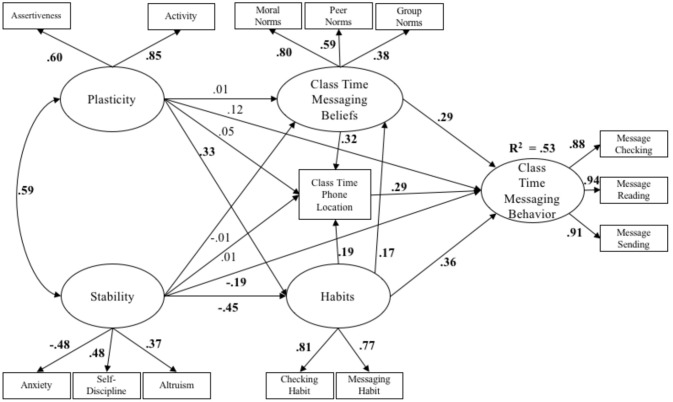
The cybernetic personality system model in the class room context (*N* = 634). All standardized path coefficients that were significant at the 0.05 level are bolded.

Figure 4 displays standardized path coefficients for the cybernetic personality system model for the context of being in class. The size and direction of effects were nearly identical to the context of eating with others, with two exceptions: Greater stability directly predicted decreased messaging behaviors, and phone-related habits directly predicted greater endorsement of normative messaging behaviors. Total, direct, and indirect effects, as well as bias corrected bootstrapped standard errors and 95% confidence intervals are displayed in Table 5.

**Figure 5:**
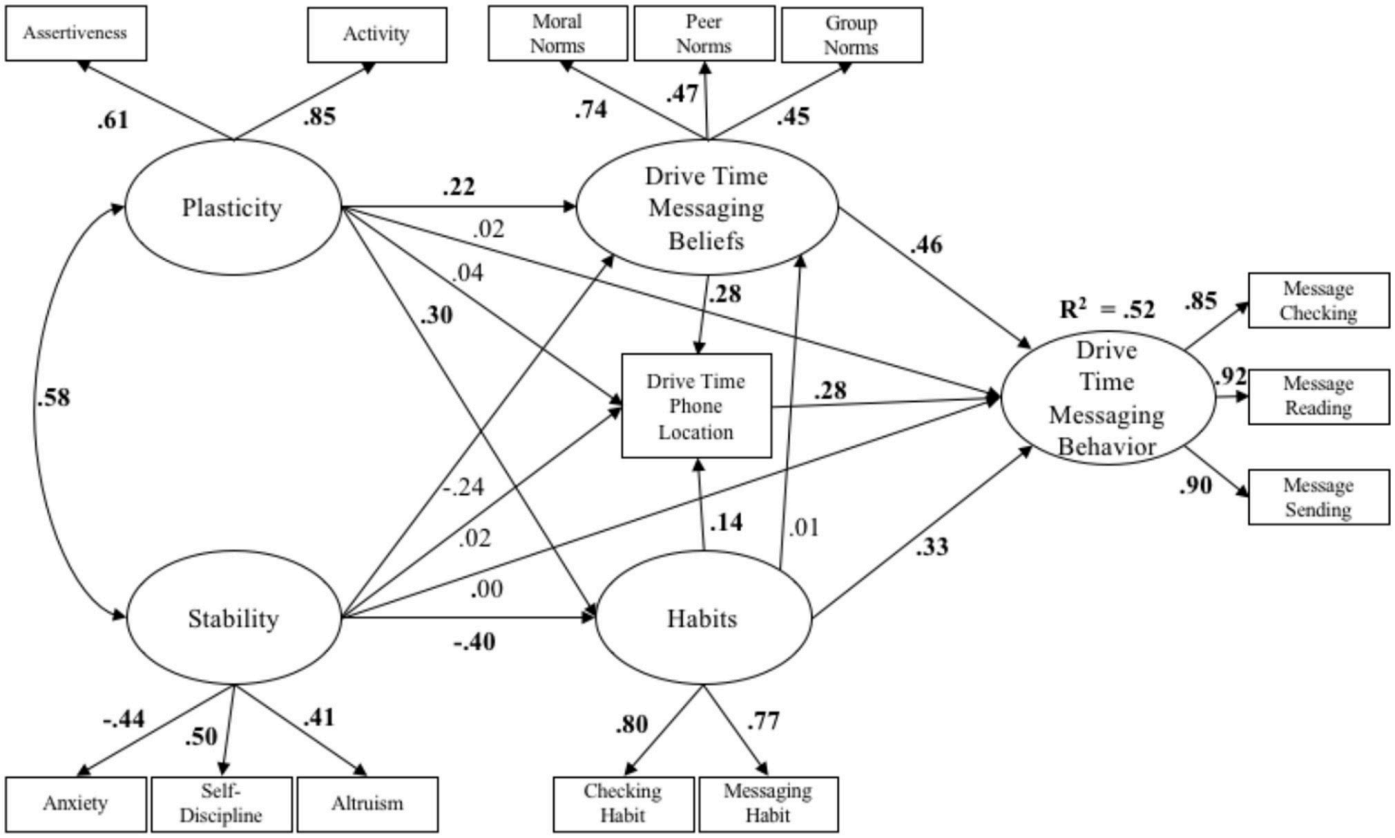
The cybernetic personality system model in the driving context (*N* = 634). All standardized path coefficients that were significant at the 0.05 level are bolded.

Figure 5 displays standardized path coefficients for the cybernetic personality system model in the driving context. Results were essentially identical to those in the context of eating with others, with one exception: Plasticity directly predicted increased endorsement of normative messaging behaviors, which in turn led to increased messaging behaviors. Total, direct, and indirect effects, as well as bias corrected bootstrapped standard errors and 95% confidence intervals are displayed in Table 5.

## Discussion

The purpose of the present study was to test and compare a sociocognitive connection model of mobile phone messaging behaviors and a cybernetic personality system model of mobile phone messaging behaviors across the contexts of eating with others, being in class, and driving. Although differences between the two proposed models were modest, the cybernetic model with theoretically-guided specific facets was selected for interpretation due to its superior comparative fit indices and its conceptual parsimony. Although alternative models were tested (with less theoretically justified facets, and with global trait scores), they either failed to converge, or had poor fit. The specification of theory-driven facets in the cybernetic model is one way in which this study contributes to the literature on personality factors and distracted mobile phone use.

Across all three contexts, greater stability (i.e., calm, self-controlled, selfless) indirectly predicted a decrease in messaging behaviors via a reduction in phone-related habits, while greater plasticity (i.e., energetic, assertive) indirectly predicted increased messaging behavior via increased phone-related habits. In addition, across contexts, greater endorsement of positive beliefs about normative messaging behaviors, greater proximity to one's phone (physical phone location), and stronger phone-related habits all directly predicted increases in messaging behavior. Only in the driving context did plasticity predict beliefs about normative messaging behaviors, and only in the classroom context did stability directly predict a reduction in messaging behaviors.

Stronger normative beliefs about messaging behavior directly predicted increased messaging behavior across all three contexts. This finding suggests that messaging social norms are important for broadly predicting messaging behaviors. That is, across a range of contexts, perceived social norms are used to gauge the appropriateness of messaging behaviors, with more positive beliefs about messaging behavior facilitating behavioral engagement with one's phone. Positive beliefs and social norms surrounding messaging behavior may increase the likelihood that one will keep one's phone accessible, and therefore ready to use as a tool for fulfilling social goals (i.e., the goals to be available to others and respond to messages promptly). The results suggest that this may be the case, regardless of how limited contextual affordances are for engaging with others. It should be noted that although normative beliefs were a strong predictor of messaging behavior across contexts, this effect was stronger in the contexts of eating with others and driving. This suggests that in the contexts of eating with others and while driving, the moral consequences of one's behavior may be especially salient in relation to other people. That is, messaging behavior while eating with others or while driving may have greater consequences for others (e.g., feelings of rejection or a collision) whereas, in class, the consequences of messaging behavior for others may be perceived as less dire (i.e., a temporary distraction). Across contexts, the results showed personality traits (i.e., low anxiety, high self-discipline, and high altruism) most strongly predicted less frequent messaging behaviors via reductions in phone-related habits.

Consistent with expectations, across contexts, the cybernetic personality system model showed greater plasticity predicted more frequent messaging behaviors via increased phone-related habits. Contrary to expectations, plasticity only predicted stronger endorsement of normative messaging beliefs in the context of driving, and plasticity did not predict less impeded phone access (physical phone location) in any context. As expected, across contexts, greater stability predicted decreased phone habits, which, as indicated by indirect effects analyses, predicted less frequent messaging behaviors. However, contrary to expectations, stability directly predicted a reduction in messaging behaviors only in the classroom context.

In line with expectations, physical phone location mediated the paths between phone-related habits and messaging behaviors, and beliefs about phone use and messaging behaviors, across all three contexts. These findings suggest that in general, where one chooses to have their phone (i.e., proximity to one's person) is driven by phone-related habits and beliefs about how normative messaging behavior is across contexts.

Taken together, these findings suggest that less frequent messaging behavior is predicted by weaker phone-related habits, which appears to be influenced by one's disposition (in particular, being calm, self-disciplined, and selfless). Beliefs about phone use, especially in contexts that have consequences for others (i.e., while eating with others and while driving), are also important predictors of messaging behaviors.

The results also suggest that behavioral norms and beliefs about messaging behaviors are strongly predictive of messaging behaviors across the contexts of eating with others, being in class, and driving, and this relationship is mediated by phone accessibility. Additionally, the proclivity to remain engaged in a primary task (i.e., stability) was strongly associated with weaker phone-related habits, which, in turn, was associated with less frequent messaging behaviors. These patterns held across all three contexts, suggesting that situational constraints may not affect messaging behaviors as strongly as one's enduring beliefs, habits, and traits. However, the context-specific patterns suggest that when consequences for others are perceived as lower (i.e., in the context of being in class), beliefs about phone use less strongly influence one's messaging behaviors while, by contrast, beliefs are particularly important for predicting messaging behaviors in contexts where one's behavior may more negatively affect others (i.e., eating with others, driving on a road shared by others). These results suggest that college students with greater plasticity and less stability are most likely to engage in messaging behaviors, regardless of their context. Furthermore, college students with more positive normative beliefs about messaging behaviors and stronger phone-related habits will be more likely to engage in messaging behaviors across contexts.

### Limitations and Implications

The present study had several limitations. Though adequate in size, the sampling strategy was focused on college students, thus findings may not be broadly generalizable. Additionally, the study design was cross-sectional, and causal claims about the ordering of study variables cannot be made. We also acknowledge that structural equation modeling represents only one analytic approach, and that alternative analytic approaches (e.g., latent profile analysis) may also be informative for assessing combinatorial trait effects. Finally, the present study was unable to examine other consequential contexts of interest that might be pertinent in college samples (e.g., while studying), and other samples, such as messaging behavior in the context of one's occupation, and/or messaging behavior in the context of one's romantic relationships (i.e., partner interactions). Longitudinal studies using daily diary methodology or experimental designs could help establish the temporal ordering of traits, beliefs, and habits for predicting messaging behaviors. Although the assessment in the present study was based in self-report, and daily diary assessments might improve the fidelity of the messaging variable assessments, ecological momentary assessments and daily diary administration might create other biases, including confounding the form of observation (via phone) with one of the behaviors under observation (phone checking).

## Conclusion

The pattern of findings in the present study suggests that across the three contexts of eating with others, being in class, and driving, beliefs about phone usage, habits, and physical phone location are robust predictors of messaging behaviors. Across contexts, personality traits that reflect stability were associated with reductions in phone related habits, and beliefs about normative phone use were also important predictors of messaging behaviors across contexts. The findings suggest that targeting both phone-related habits and normative beliefs about phone use may be the most effective points of intervention for attempting to reduce distracted messaging behaviors across all three contexts. Previous research on interventions aimed at reducing alcohol consumption have shown that using motivational interviewing, and in particular, highlighting the discrepancies between perceived norms and actual behavior, can effectively reduce problematic behavior (Larimer and Cronce, 2007), and this same principle might be applied to texting while eating with others, being in class, or driving. If college students come to realize that their messaging behavior (i.e., texting while eating, being in class, or driving) is less normative than previously thought, then this may lead to an adjustment of internalized social norms and beliefs about messaging that could ultimately lead to reduced messaging behavior in consequential contexts. In addition, across contexts, the strong negative link between stability and messaging habits suggests that interventions should focus on behaviors that are associated with self-discipline, anxiety, and altruism, which strongly predict phone habits. For example, identifying specific traits and attempting to enhance (i.e., for self-discipline) or reduce (i.e., for anxiety) behaviors associated with those traits may lead to reduced messaging habit formation, and ultimately, less messaging behavior that constitutes primary task disengagement.

## Author Contributions

JB collected the second wave of data, conceptualized the sociocognitive connection model, conducted all analyses, drafted the manuscript, and approved the content for publication. TB conceptualized the cybernetic self-regulatory model, edited the manuscript, supervised data analysis, and approved the content for publication. JH collected the first wave of data, contributed to conceptualizing the sociocognitive connection model, provided draft edits, and approved the content for publication.

### Conflict of Interest Statement

The authors declare that the research was conducted in the absence of any commercial or financial relationships that could be construed as a potential conflict of interest.

## Footnotes

^1^Alternative sociocognitive and cybernetic models that substituted alternative facets for each Big Five trait were also analyzed to test whether the six selected facets of assertiveness and activity (for extraversion), altruism (for agreeableness), ideas (for openness), self-discipline (for conscientiousness), and anxiety (for neuroticism) produced better fitting models than available alternative facets for each trait (i.e., compliance for agreeableness, aesthetics for openness, orderliness for conscientiousness, and depression for neuroticism). Additionally, a second set of alternative models was tested using the global trait scales for the Big Five (i.e., extraversion, agreeableness, conscientiousness, neuroticism, and openness) substituted in place of facets. These alternative sets of models either did not converge or demonstrated poor to mediocre model fit.
